# Evaluating the Influence of Normalisation Procedures on a Negative Selection Algorithm to Enhance Damage Detection

**DOI:** 10.3390/s26113492

**Published:** 2026-06-01

**Authors:** Alberto Barontini, Maria-Giovanna Masciotta, Luís F. Ramos, Paulo Amado-Mendes, Paulo B. Lourenço, Giuseppe Brando

**Affiliations:** 1 Department of Engineering and Geology, “G. d’Annunzio” University of Chieti-Pescara, 65127 Pescara, Italy; alberto.barontini@unich.it (A.B.); g.masciotta@unich.it (M.-G.M.); giuseppe.brando@unich.it (G.B.); 2 ISISE, Department of Civil Engineering, University of Minho, 4800-058 Guimarães, Portugal; pbl@civil.uminho.pt; 3 ISISE, Department of Civil Engineering, University of Coimbra, 3030-788 Coimbra, Portugal; pamendes@dec.uc.pt

**Keywords:** damage identification, vibration-based structural health monitoring, bridge management, dynamic identification

## Abstract

Damage detection is a critical component of Structural Health Monitoring (SHM) strategies. Data-driven machine learning methods are widely employed for this purpose. However, their effective application to the management of structures and infrastructure requires addressing key challenges, in particular the limited knowledge during training of how damage affects monitored features. Although one-class classification algorithms may be adopted, their performance strongly depends on the appropriate definition of their components and the tuning of their parameters. When this optimisation is performed using undamaged data only, it may result in classifiers that are insensitive to small-scale damage. Within this context, the present study investigates the sensitivity of damage detection performance of a Deterministically Generated Negative Selection Algorithm to feature scaling and two intrinsic algorithm parameters. A novel strategy for generating artificial damaged data to support parameter tuning is proposed and evaluated against alternative approaches. A wide range of parameter values is explored, considering multiple pairs of monitored features as detection spaces, and four feature scaling methods are compared. Feature scaling is a fundamental aspect of classification problems. Thus, the main findings may be generalised to other machine learning algorithms for damage detection. To ensure full control over the monitoring data and underlying phenomena, a numerical case study is adopted. A replicable framework for generating controlled yet realistic structural monitoring data is presented. The simulated monitoring captures the natural frequencies of a bridge, accounting for temperature effects, and incorporates three damage scenarios: one diffuse and two localised at critical locations. The analysis highlights the importance of conducting anomaly detection in feature spaces where damage affects each feature differently. Pairing a damage-sensitive feature with one unaffected by damage (e.g., temperature) may be advantageous when damage effects are not known a priori. Although suboptimal, the proposed strategy for generating damaged data for parameter tuning outperforms approaches based solely on undamaged data. The identified parameter trends suggest that small detector radii and relatively short censoring distances improve algorithm performance across all normalisation strategies and damage scenarios. The results show that feature scaling has limited influence for large damage extents but becomes critical for the early detection of minor damage. Z-score normalisation provides the best overall balance between false negatives and false positives, whereas methods using denominator multipliers smaller than one provide higher True Positive Rates for small damage extents.

## 1. Introduction

Within the field of Structural Health Monitoring (SHM), several automated condition assessment and damage identification (DI) strategies have been developed [1,2,3]. This field is characterised by a well-established hierarchy organised into the following levels [3]: (1) detection of damage occurrence; (2) localisation within the structure; (3) classification of damage type; (4) quantification of damage extent; and (5) prediction of the remaining service life. This work focuses on the first level. DI strategies require the continuous or periodic sampling of measurable quantities from both the system itself and its environment through suitable sensors deployed on the structure. The processing of these observations allows the extraction of damage-sensitive synthetic indicators that provide information on the system’s fitness for purpose under operating conditions [4,5]. Indicators derived from vibration monitoring have become widely used features for DI purposes [2,6]. Critical structures and infrastructures, such as bridges, have increasingly been the subject of long-term monitoring campaigns, including vibration-based monitoring. In this context, automated operational modal analysis has emerged as a widely adopted strategy for identifying dynamic properties, such as natural frequencies, mode shapes, and damping ratios, using only ambient excitations (e.g., traffic, wind, or microtremors). Since it does not require controlled input forces, this approach is particularly well suited for in-service assessment of structures. Several algorithms have been developed to implement such strategies [7,8,9,10].

The effectiveness of DI strategies and the level achieved within the aforementioned hierarchy strongly depend on the characteristics of the sensors, the strategic optimisation of their placement, and the implementation of robust numerical techniques to post-process and interpret monitoring data [11,12,13,14]. The present work focuses on the latter component. Data processing may be conducted according to two main approaches, depending on whether knowledge of the physical behaviour of the system and the underlying phenomena is incorporated. Data-driven approaches, either fully black-box or partially incorporating physical knowledge, as in the so-called physics-informed machine learning field [15,16,17], have attracted particular attention [18]. These approaches require little or no prior information about the system and its properties, thereby avoiding common issues arising from large uncertainties in geometry, structural details, material properties, loadings, and boundary conditions, which are typically required for the development of reliable numerical models [19,20,21].

Mathematically, damage detection can be formulated as a one-class machine learning classification problem [22,23]. Within this framework, each new record is processed by a classifier that assigns either a negative or a positive label, depending on whether it is recognised as normal or anomalous. An anomaly, particularly if confirmed over consecutive acquisitions, is then associated with the potential emergence or progression of damage [24]. The classifier is trained under conditions typical of engineering systems, where information is generally available only for a reference state. Only a limited number of machine learning algorithms are suitable for this type of problem. Among them, the present work focuses on Negative Selection Algorithms (NSAs) [25,26], a relatively inexpensive method that has been extensively explored in several computer science applications [27,28] but remains largely underexplored for anomaly detection in mechanical, aerospace and civil engineering.

As further detailed hereafter, the rationale for this work is based on three major challenges currently encountered in the application of machine learning to damage detection in civil structures: (1) the scarcity of realistic damaged data and limited representativeness of existing benchmarks across different structures and environmental conditions [29,30,31]; (2) consideration of the intrinsic characteristics of real-world problems, including limited knowledge of system behaviour and the need to train anomaly detection algorithms using only samples from the reference condition, with scarce or no labelled damage data available [30,32,33,34]; and (3) the trade-off between false alarm rates and sensitivity to small damage extents in the context of early warning applications [35,36,37]. These issues are not intrinsic to the use of NSA algorithms, as they are potentially shared by most machine learning approaches for anomaly detection in civil engineering structures and infrastructures. Although the present study focuses on NSAs, it seeks to address these limitations through scalable and generalisable solutions by: (1) introducing a replicable strategy for generating realistic data for algorithm development and testing using simple yet accurate numerical models; (2) assessing the influence of algorithm parameters on detection performance and proposing a parameter calibration strategy based on the limited initial knowledge available from samples of a single reference condition; and (3) investigating a relatively unexplored approach to improve responsiveness by performing classification in feature spaces obtained through different scaling methods.

Most existing studies adopting NSAs have relied on high-dimensional feature vectors, typically entire signals processed in either the time or frequency domain [38]. For example, de Almeida et al. [39] applied an NSA-based approach to detect anomalies in impedance data from a PZT-based monitoring system on a composite plate, considering both temperature variations and progressive delamination. Delo et al. [40] used an NSA to analyse transmissibility functions from a thin-section box fuselage under different damage scenarios, simulated by adding concentrated masses and tested across varying operating conditions. However, the use of long signals can negatively affect classification performance due to the “curse of dimensionality”, requiring careful feature encoding or pre-processing. To address this, Li et al. [41] transformed raw microtremor acceleration data through symbolisation before applying a tailored NSA. Alternatively, Shi and Yu [42] reduced the dimensionality by considering as a feature the energy distribution of the signal decomposed by a wavelet transform at different frequency bands. Earlier work, by Li and Ren [43], employed pseudo-random real-valued detectors to identify anomalies in the first four natural frequencies of a simply supported steel beam, accounting for temperature effects through the temperature-dependent Young’s modulus and random effects through additive noise. While the reported results are promising, the selection and tuning of algorithm parameters and hyperparameters are frequently neglected in the literature, highlighting the need for further investigation. Additionally, critical issues of NSAs, partly shared with other soft-computing approaches for damage detection, have been identified by the authors in [24], particularly concerning detection readiness and its trade-off with false alarm rates. The authors have previously addressed these issues by developing and testing a Deterministically Generated Negative Selection Algorithm (DGNSA) using numerical, laboratory, and field-testing datasets [44], as well as by implementing replicable strategies aimed at improving confidence in classification outcomes, balancing early warning capability and false positives, even with limited training data and small damage levels [45,46].

Building upon these foundations, the present study provides a systematic investigation of the algorithm’s capabilities and limitations under more demanding conditions, with the aim of improving its robustness and practical applicability. In particular, the version analysed herein incorporates an enhanced censoring strategy and examines in greater detail the influence of the algorithm parameters, providing new insights into their role in achieving reliable coverage of the boundary between normal and anomalous data. In addition, different normalisation strategies, as reported in [47], are critically compared to evaluate their impact on early-stage damage detection. Normalisation is a critical step in machine learning, as appropriate scaling can improve both convergence and classification performance. Moreover, as it concerns the representation of input data within the feature space rather than the specific algorithm, the findings of this study are expected to be transferable to other soft-computing methods for damage detection.

The development and validation of damage identification tools, as undertaken in this work, require the availability of extensive datasets, ideally derived from controlled and reliable sources. This is essential, as uncontrolled uncertainties or errors in the measurement chain may compromise the interpretation of the underlying phenomena and, consequently, lead to a biased assessment of algorithm performance.

Several factors can influence SHM data, including operational and environmental variations and progressive or sudden damage, as well as sensor faults and malfunctions [46,48,49,50,51]. Real-world datasets provide valuable opportunities to capture these effects in a natural and realistic manner. However, their inherent variability makes them difficult to control and to isolate specific phenomena during the development phase of monitoring tools. In contrast, artificial datasets allow full control over data quality, patterns and trends, but require careful design to ensure that simulated variations are realistic and representative, thereby providing a reliable testbed for algorithm evaluation. Key challenges in the development of numerical case studies for SHM are discussed in [29], which also presents an overview of established benchmark models.

Within this context, a further objective of this work is to provide a simple yet realistic and informative case study, supported by replicable strategies for generating numerical benchmarks suitable for SHM applications.

The remainder of the paper is organised as follows. Section 2 describes the overall damage detection framework, including the algorithm components and parameter setting approach. The case study is introduced in Section 3, outlining the replicable strategy adopted for its generation and presenting the simulated monitoring data. The main results are presented and discussed in Section 4. Finally, conclusions and directions for future research are provided in Section 5.

## 2. Methodology

The proposed damage detection strategy builds upon a deterministically generated version of the NSA developed by the authors. NSAs are a family of algorithms inspired by the behaviour of the human immune system based on a common conceptual framework improved over recent decades, with applications across multiple fields [25,27,52]. NSAs perform anomaly detection by processing damage-sensitive features after scaling them to a defined feature space. Classification is carried out using a set of so-called detectors, which are trained to cover the nonself region of the feature space, namely the region where features associated with anomalous structural behaviour are expected to lie. Conversely, self samples, corresponding to normal conditions, occupy the remaining portion of the space. For a thorough analysis of the NSA theoretical background the reader can refer to [53,54,55]. This framework comprises three main consecutive stages: (1) representation; (2) censoring; and (3) monitoring.

**Figure 1:**
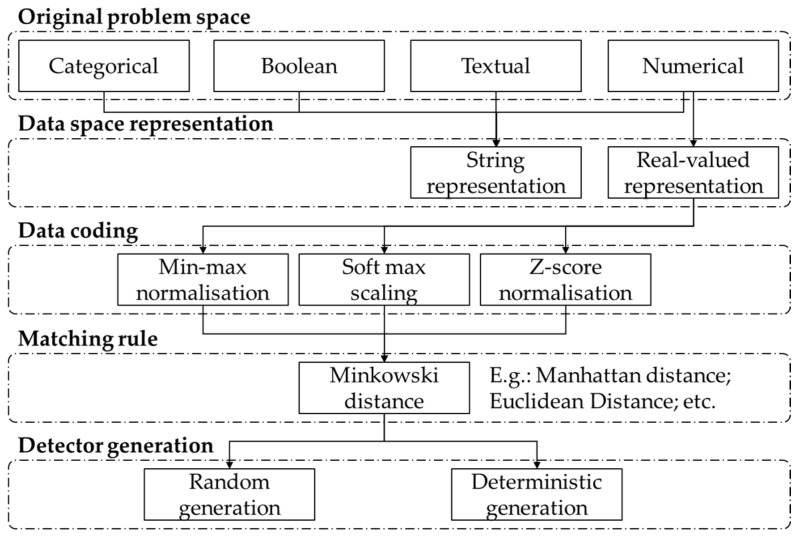
Higher level components of NSAs with common options and hierarchical organisation.

Preliminary operations, in the representation stage, include an informed definition of the higher-level components of the algorithm as shown in Figure 1, namely [55]: (1) the original problem space; (2) the data space representation; (3) the data coding; (4) the matching rule; and (5) the detector generation/censoring strategy. These components are ordered in a top-to-bottom way as each one limits the setting of the following. First applications have formulated the problem in binary spaces, resorting to string representations. However, in this low level representation, the encoding process does not reflect the distance among the data in the original space and different concepts of distance and similarity must be introduced, limiting the interpretability of the results [53,55]. Additionally, string representation suffers from scalability issues.

In SHM applications, it may be more intuitive to define the problem in a numerical space encompassing all possible real values of the monitored features. However, as in most machine learning problems, the original input domain is rarely used directly as the feature space. Instead, features are typically encoded by rescaling their values into a predefined range. In the present study, as commonly done, the range adopted is [0, 1], thus, the feature space is *U* = [0, 1]^*n*^, where *n* is the number of the features investigated. This rescaling, or normalisation, can improve both the learning speed and the overall performance of the algorithm.

While, in previous works, the authors have considered a single preferred normalisation method, namely the min–max normalisation, in the present study, three normalisation algorithms, discussed in [47] for applications in other fields, are compared to assess how the choice can affect the damage detection performance. Adopting the nomenclature presented in [47], these are: (1) min–max normalisation; (2) soft max scaling; and (3) Z-score normalisation.

Given a generic feature *m*, min–max normalisation (MMN) maps each value *x*_*mi*_ of the feature *m*, measured at time interval *i*, into a new interval ranging between 0 and 1 according to the following:

(1) $${\overset{̿}{x}}_{mi}=\frac{x_{mi}-\min\left(x_{mi}\right)}{\max\left(x_{mi}\right)-\min\left(x_{mi}\right)},$$

where the upper and lower bounds of the range are defined based on the data available for the training. This is necessary to prevent rescaling upon new acquisitions but introduces the risk of “out of range” records, namely new elements, whose values exceed the predefined bounds. A possible solution consists in setting specific rules leveraging case-specific knowledge when available. A larger range, commonly a 20% increase, is often considered. Samples falling outside the expected range may either be classified as anomalous, if the bounds reasonably represent the maximum variability of the feature, or left unclassified when out-of-range values may arise from previously unseen but normal conditions. This distinction is particularly important for operational and environmental variations, as extreme operating or weather conditions may produce values well beyond the known range without necessarily indicating the presence of damage. In this study, the upper and lower bounds of each feature are defined by increasing and decreasing the observed maximum and minimum values by 20% of the original range. This results in an expanded feature space that fully encloses the training data and is subsequently covered by detectors, thereby increasing the likelihood that new samples outside the training domain are identified as anomalous, unless they fall within a narrow boundary region left during the censoring stage between the training data and the detector coverage.

Thus, the impact of out-of-range values does not depend solely on the variability of a single feature, but rather on the combined effect of all features in the scaled space, as well as on the algorithm parameters governing the distribution of the detectors. Consequently, assessing a priori the likelihood that such samples lead to false positive outcomes is inherently complex. For this reason, rather than relying on probabilistic estimates, it is generally more effective to adopt robust and well-established strategies in the definition of the training set. Among these, ensuring that the training data capture at least one full cycle, but preferably more, of the environmental and operational variables influencing the damage-sensitive features is of primary importance, as it significantly reduces the probability of encountering unseen conditions during the monitoring phase [56,57,58,59,60].

Soft max scaling (SMS) comprises a two-stage process. In the first stage, the value of the attribute *m* is mapped onto a range based on the distance between the mean value ${\bar{x}}_{m}$ and the standard deviation *σ*_*m*_ of the training data.

(2) $${\overset{ˇ}{x}}_{mi}=\frac{x_{mi}-{\bar{x}}_{m}}{\lambda \left(\frac{\sigma_{m}}{2\pi }\right)},$$

Here, λ=3 is adopted. In the second stage, the new values are mapped onto the interval [0, 1] through the function:

(3) $${\overset{̿}{x}}_{mi}=\frac{1}{1+\exp\left(-{\overset{ˇ}{x}}_{mi}\right)},$$

Similarly, the z-score (ZS) normalisation is a two-stage process, where the first stage presents an alternative definition of the range based on the mean value ${\bar{x}}_{m}$ and standard deviation *σ*_*m*_:

(4) $${\overset{ˇ}{x}}_{mi}=\frac{x_{mi}-{\bar{x}}_{m}}{\lambda \sigma_{m}},$$

Here, two values are adopted for λ, namely 3 (hereafter 3ZS) or 1 (hereafter 1ZS). This latter case corresponds to the standardisation of the feature, one of the most widely used scaling methods in machine learning [61,62], whereas the former may be associated with the three-sigma rule [63]. The second stage is equivalent to the previous method, according to Equation (3). SMS and ZS do not suffer from the “out of range” problem. Moreover, it is evident that the method hereafter referred to as SMS can be interpreted as a 0.48ZS approach, where 0.48 is the multiplier applied to the standard deviation in the denominator. Accordingly, the results of the analyses may be interpreted as an assessment of the sensitivity of the damage detection performance to the parameter λ, compared with the reference MMN approach.

Following the encoding, a definition of the matching rule and the detector generation strategy is needed. The matching rule consists of a distance measure and a threshold value. The detectors should classify as anomalous the records that fall sufficiently close to them without requiring a perfect coincidence. Matching rules may change between the censoring and the monitoring stage to account for the limited knowledge in the training set. In the present work, the Euclidean distance is used as the measure and the threshold, hereafter called the detector radius, is optimised through a parameter setting strategy. Moreover, a different threshold is introduced for the censoring stage, whose value is subjected to a parameter setting procedure as well.

The detectors may be generated either randomly or deterministically. In low-dimensional spaces, deterministic generation can provide a straightforward means of constructing the detector set while ensuring full coverage of the feature space, although at a higher computational cost. Deterministic generation schemes for real-valued NSAs have been explored in several fields [64]. In this work, an enhanced version of the generation and censoring scheme originally proposed in [44] is presented. According to it, the detector set is initialised as a regularly spaced grid in the feature space.

However, such exhaustive coverage becomes increasingly impractical as dimensionality grows. This scalability issue, common to NSA formulations in general [65], is, in the present approach, associated with the exponential increase of the number of detectors required to cover an *n*-dimensional feature space using a regular grid with *m* points per dimension. The computational burden is further exacerbated by the fact that each detector is defined by *n* coordinates. Thus, in high-dimensional settings, both the cost of generating and processing the grid and the memory required to store it rapidly become prohibitive.

On the other hand, although higher-dimensional spaces may appear advantageous, as they can incorporate the sensitivity of a greater number of features, it has been shown that in such spaces even large training datasets may become sparse and poorly representative [66]. This sparsity implies that a substantially larger number of samples would be required to characterise the self state adequately, which is rarely feasible in practice. Moreover, the self space may become extremely small relative to the nonself space. Under these conditions, negative selection becomes less effective, as it attempts to describe highly complex or excessively large regions. This has been observed in previous studies involving different problems and encoding strategies [67,68,69]. The challenge of covering a hypercubic space with hyperspheres in *n*-dimensional feature spaces was discussed in [66]. That study demonstrated that, for small detector radii, the volume of the hypersphere tends towards zero as *n* grows, while the volume of the corresponding hypercube becomes concentrated near its corners, thereby compromising efficient coverage of the nonself space. For these reasons, a two-dimensional feature space is adopted in this work to mitigate the computational burden associated with high-dimensional initialisation. Furthermore, the use of multiple two-dimensional feature spaces in parallel is proposed as a strategy to exploit different sensitivities to damage.

**Figure 2:**
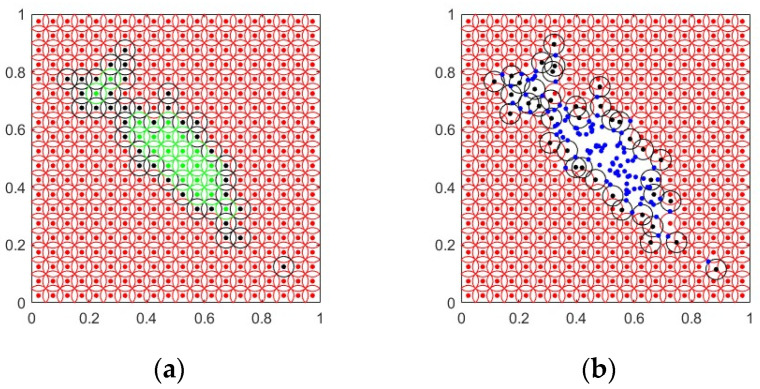
Censoring stage, dimensionless unitary [0, 1]^2^ feature space: (**a**) regular detector grid (red), with internal detectors identified for removal (green) and boundary detectors selected for relocation (black); (**b**) regular detector grid (red), relocated boundary detectors (black) compared with the self samples (blue).

For the generation, the only required input is the spacing *b* between detector centres in the two-dimensional space. To ensure uniform spacing, the points are evenly distributed, with the first and last points located at a distance of *b*/2 from the boundaries of the unit space. Given this spacing, the detector radius required to fully cover the space around the centres, while minimising overlap, is $r_{det}=\sqrt{b^{2}/2}$. In the feature space, detectors can be represented as circles, with centres defined by their coordinates and radii equal to the matching threshold (Figure 2).

Following initialisation, the censoring stage encompasses the training of the machine learning algorithm to produce the optimised classifier. This involves comparing the regular grid of detectors with the training data, which consists solely of self elements. To this end, the matching threshold, hereafter called the censoring distance, is defined as a value equal to or greater than the detector radius. This accommodates the possibility of encountering self samples located near boundary regions during monitoring, thereby preventing overly aggressive classification behaviour.

A temporary set is used to collect all detectors that match at least one self element (Figure 2a). From this set, a novel strategy, here adopted, identifies and remove detectors that have four neighbouring detectors at a distance equal to *b* (i.e., fully surrounded by adjacent detectors), highlighted in green in Figure 2a, leaving only the boundary detectors. These boundary detectors (highlighted in black in Figure 2) are then iteratively displaced away from the nearest self element (Figure 2b), following the strategy proposed in [70]. To this end, a maximum number of iterations *t* is defined. At each iteration *i*, the detector *d_j_* is moved away from the closest self identified at the same iteration *s_k_*, in the direction $dir=\frac{d_{j}-s_{k}}{distance(d_{j},s_{k})}$ by a step size equal to $\eta_{i}=\eta_{0}\exp\left(-i/t\right)$. The process ends for a given detector when it lies beyond the censoring distance from any self sample or when the maximum number of iterations is reached. The next detector is then processed. Finally, the boundary detectors, in their updated positions, are incorporated into the regular detector set.

Among the algorithm parameters, the maturity age *t* and adaptation rate $\eta_{0}$ are kept constant at 15 and 0.005, following recommendations from the literature (e.g., [71,72]). Although the optimisation of these parameters is rarely investigated, pilot tests and sensitivity analyses performed by the authors on other case studies, such as [73], have shown that they exert a limited influence on overall performance. In contrast, the detector radius and the censoring distance are optimised using a validation dataset. A critical limitation of one-class classification is the absence of nonself samples in the validation set. Parameter optimisation based solely on self data has been shown, in binary classification contexts, to produce an imbalanced trade-off between false positives and false negatives, as reducing one increases the other, and vice versa [24]. While previous studies have addressed this issue by generating artificial outliers from the marginal distributions of the features [45], a novel alternative strategy is adopted in the present work. Specifically, nonself validation samples are generated by translating self validation samples along the narrow direction of the data distribution, thereby producing a dataset that overlaps the boundary regions. To this end, if the covariance between the two features is negative, samples are translated in the positive direction of both features, and in the negative direction of both, by a multiple of their respective variance. Conversely, if the covariance is positive, samples are translated in opposite directions: positively along one feature and negatively along the other, and vice versa. Clarifying examples are provided within the case study application.

This validation stage aims at identifying the best classifier according to a well-established performance metric. Based on the authors’ experience, the area under the Reception Operating Characteristic (ROC) curve, hereafter called ROCAUC, is adopted due to its lower sensitivity to the size of self and nonself samples. The advantages and limitations of performance metrics in binary classification are discussed in [74,75,76] and specifically for the DGNSA in [44]. In the present work, two additional metrics are used to assess the classifier behaviour, namely the False Positive Rate (FPR) and the True Positive Rate (TPR). These metrics are related through the following relationship:

(5) $$ROCAUC=\frac{1}{2}\left(TPR+1-FPR\right)\;TPR=\frac{TP}{TP+FN}\;FPR=\frac{FP}{TN+FP}$$

The optimised classifier, in each bidimensional feature space, composes the offline initialised detector sets.

Finally, the monitoring stage performs the actual anomaly detection. In SHM problems, this involves analysing new observations from the monitored system, projected onto the feature space, using the trained detector set. Detectors that match these observations classify them as anomalous. Reflecting the decentralised and distributed nature of the immune system, multiple autonomous detector sets can be employed to assess different components of the system or distinct combinations of features. In particular, deploying multiple classifiers in complementary feature spaces enables parallel monitoring and has been shown to improve damage detection by exploiting the varying sensitivity of different features [45]. This is further confirmed hereafter in the application to the case study, considering different damage scenarios.

## 3. Case Study

An ad hoc, realistic benchmark case study was developed. The main steps involved in its generation, along with its key characteristics, are presented below to ensure the reproducibility of the results. The case study consists of a finite element model of a three-span reinforced concrete bridge, incorporating simplifications based on numerical benchmarks previously developed by the authors for SHM applications, including dynamic identification algorithm development [77] and optimal sensor placement [78], while being inspired by the Z24 Bridge, a well-established SHM benchmark [79,80,81]. A two-dimensional analysis is performed, focusing on the longitudinal and vertical structural responses. The introduced simplifications aim to balance data realism with computational efficiency in generation and management, while enabling the definition of challenging conditions to assess algorithm performance.

**Figure 3:**
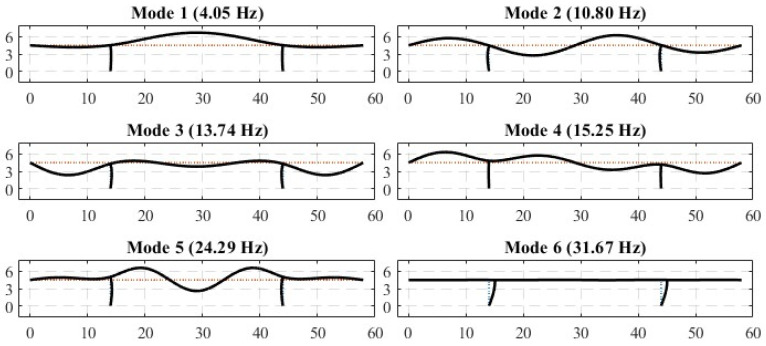
Numerical vibration modes of the case-study bridge.

The case study is implemented in a MATLAB environment. The side spans are 14 m long, while the central span is 30 m. A hollow box cross-section measuring 8 m × 1.1 m, with a thickness of 0.35 m, is adopted for the deck. The two piers feature, instead, a 1 m × 1 m square section and a height of 4.5 m. The structure is discretised using 0.5 m long Euler–Bernoulli plane beam elements with constant geometry and material properties, accounting for axial deformation. Each element consists of two nodes, each with three degrees of freedom, resulting in a total of 134 elements, 135 nodes and 405 degrees of freedom. The bridge is pinned at the base of the piers and at the ends of the side spans. The reference material density is set to 2500 kg/m^3^, and the Young’s modulus is 34 GPa at the reference temperature of 20 °C. Only the linear behaviour of the system is analysed, and no additional masses or applied loads are considered. The first six vibration modes, obtained through eigenvalue analysis of the reference model, are presented in Figure 3.

Mode 1 involves a single curvature symmetric vertical bending of the central span. Mode 2 presents a second bending of the central span with counterphase bending of the lateral spans. Mode 3 is mostly involving the lateral spans which deform in phase, while Mode 4 combines the lateral span bending with a double-curvature deflection of the central span and a noticeable vertical response of the piers. Mode 5 features a three-curvature bending pattern of the central span, while, finally, Mode 6 is characterised by a longitudinal response of the bridge.

**Figure 4:**
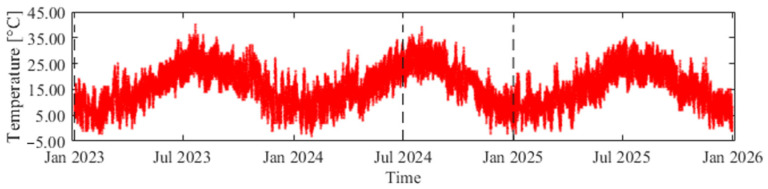
Temperature time series for the simulated monitoring. Dashed lines mark the end of the training period (1 July 2024) and validation period (1 January 2025).

Experimental studies suggest that temperature variations affect Young’s modulus, which plays a key role in the variation of natural frequencies, while generally having a smaller influence on mode shapes and a more uncertain effect on damping ratios [82,83,84]. The temperature–Young’s modulus relationship for the concrete suggested by Model Code [85] is used to induce environmental variations in the data:

(6) $$E=E_{20}\left(1.06-0.003T\right),$$

where $E_{20}$ is the Young’s modulus at 20 °C and T is the temperature. The time history of the temperature is obtained for three years of hourly acquisitions targeting typical Mediterranean coastal weather. Standard meteorological station data for the city of Pescara has been used for the present case study. Commonly, online repositories only provide a single maximum and minimum value per day. Therefore, the hourly values are obtained by interpolating these records. The final temperature series is presented in Figure 4.

In addition to this macroscopic effect, the relationship between temperature and natural frequencies has been shown to exhibit non-linear behaviour, particularly in the presence of freezing conditions [86]. Variations in dynamic properties induced by temperature are otherwise generally limited, unless thermal expansion produces changes in boundary conditions or induces additional stress states [86,87,88,89]. These phenomena are neglected in the present case study. Other sources of variations arising from factors such as temperature gradients, airflow, and solar exposure [90,91,92], together with measurement noise and processing uncertainties [93], are not explicitly modelled in the present work although they could be introduced following adequate recommendations [29,45], provided that a detailed characterisation of the environmental conditions is available. However, a simplified strategy is adopted to account for common sources of noise and uncertainty in the real structure, as well as in the measurement chain and data processing. Accordingly, the density and Young’s modulus are not treated as deterministic properties. Instead, for each mesh element, these parameters are sampled from Gaussian distributions with mean values equal to the reference values, at the corresponding temperature, and a coefficient of variation of 3%. This percentage is determined through a case-specific assessment of the final feature distributions and the sensitivity of the DI process to the induced variation. Although material properties in real structures may exhibit greater variability and potentially non-Gaussian distributions, the variability introduced in the present study is intended only to represent small fluctuations associated with local environmental and operational effects that are not explicitly modelled. Therefore, the adopted approach aims to capture minor deviations from the simulated phenomena rather than large-scale material variability, which would be unrealistic for a single structure subjected to relatively consistent conditions over time. The aim is to balance the need to obtain realistic fluctuations in the natural frequency [84,94,95,96], as well as in the scatter plots of the frequency and the frequency–temperature relationships, with the requirement to provide a sufficiently challenging testbed for the algorithm.

Overall, three damage scenarios are considered, targeting different realistic conditions. The first scenario (D1) consists of a diffuse deterioration corresponding to a 0.5% reduction of the Young’s modulus of the entire structure every 2 months. Scenario 2 (D2) introduces a concentrated deterioration at the base of both piers along the 2 m from the ground, with a reduction of 5% of the Young’s modulus every 2 months. Finally, the third scenario (D3) is a concentrated deterioration in the deck around the intersection on the right pier, 2 m on each side of the pier, with a reduction of 5% of the Young’s modulus every 2 months. These scenarios could reflect the impact of corrosion, either diffuse or localised at critical locations, due to water flow or inadequate water management, as well as the consequence of other mechanisms such as the settlement of the right pier (D3). To ensure manageability of the data for generation, storing and processing, these phenomena are accelerated to take place within one year. Nonetheless, the stepwise increase of the extent ensures the assessment of the readiness of the algorithm in detecting damage at the earliest stage.

**Figure 5:**
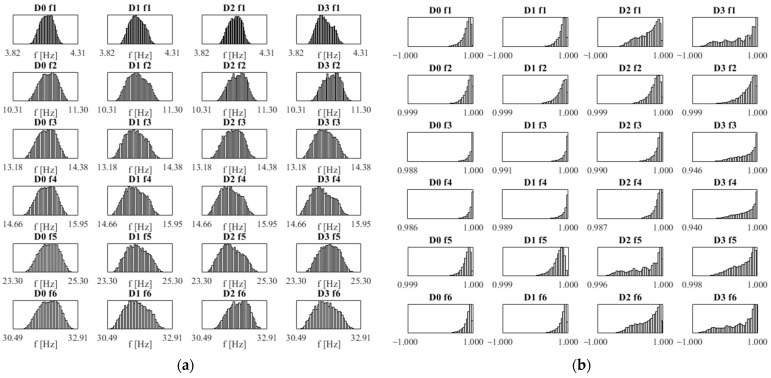
Distribution of the first six natural frequencies (**a**) and MAC (**b**) in the undamaged (D0) and damaged (D1–3) scenarios.

**Figure 6:**
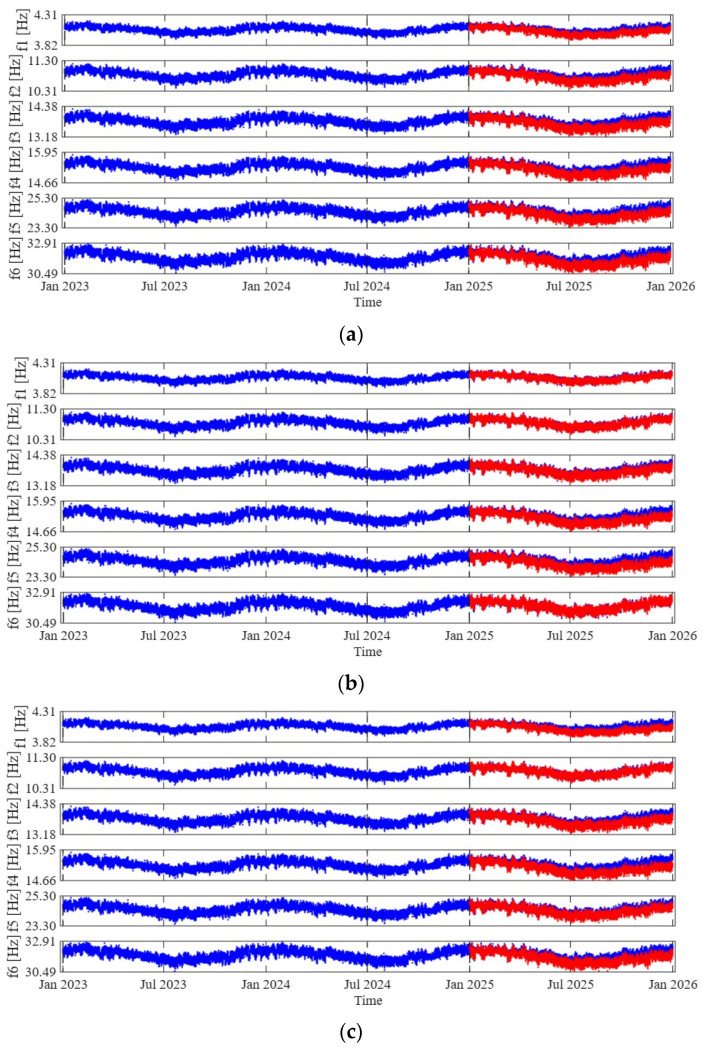
Natural frequencies, time series, undamaged samples in blue, and damaged samples in red: (**a**) D1 scenario; (**b**) D2 scenario; and (**c**) D3 scenario. Dashed lines mark the end of the training period (1 July 2024) and validation period (1 January 2025).

**Figure 7:**
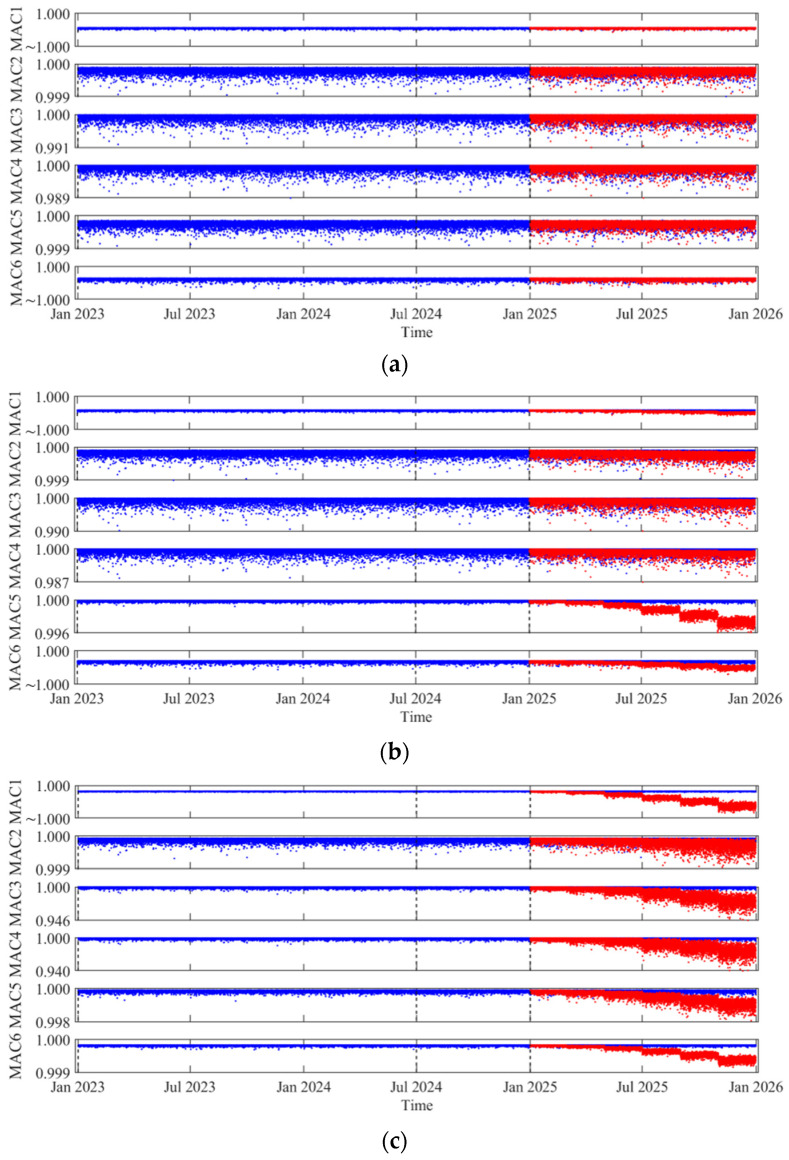
MAC, time series, undamaged samples in blue, and damaged samples in red: (**a**) D1 scenario; (**b**) D2 scenario; and (**c**) D3 scenario. Dashed lines mark the end of the training period (1 July 2024) and validation period (1 January 2025).

The results of the eigenvalue analyses for all scenarios are reported in Figure 5a for the natural frequencies and in Figure 5b for the Modal Assurance Criterion (MAC), calculated with respect to the reference undamaged state at 20 °C. The MAC is adopted here as a synthetic metric to quantify variations in mode shapes. In Figure 6 and Figure 7, time series of the natural frequencies and MAC values over the monitoring period are shown.

Damage induces a downward shift in the distribution of natural frequency samples. For scenario D1, this trend is consistent across all modes, whereas for D2 and D3 the modal sensitivity to damage varies. As expected, D1 produces a diffuse reduction in structural stiffness, with negligible influence on mode shapes. Accordingly, the MAC variations in D1 remain comparable to those observed in the undamaged configuration due solely to parametric uncertainties. Mode shapes 3 and 4 are more sensitive to both uncertainties and localised deck damage (D3). Localised damage to the piers (D2) mostly affects modes 1, 4, and 5, considering the mode shapes and frequencies. To ensure consistency in automated damage detection, a MAC-based selection criterion is adopted to retain the modes exhibiting the highest similarity to a reference shape. This procedure is simplified compared with real-world operational modal analysis, as exact modal parameters can be directly computed when generating artificial monitoring samples. A pilot study carried out to define appropriate damage levels showed that the larger reductions in the Young’s modulus in damage scenario D2 were prone to causing interchange between closely spaced modes, particularly modes 6 and 7. For this reason, smaller damage levels were selected to avoid abrupt modal changes and ensure a reliable assessment of the damage detection algorithms, with no mode swapping observed in the final scenarios, as confirmed by the MAC values presented in Figure 5b and Figure 7. The maximum average frequency shift of each scenario is presented in Table 1. This is estimated under the reference conditions of 20 °C, assuming no random variation in the Young’s modulus or density, in order to provide an unbiased assessment of the damage effect.

## 4. Results and Discussion

Damage detection is performed using five features, consisting of the temperature and the natural frequencies corresponding to modes 1, 4, 5, and 6. The aim is to include an extrinsic variable representative of environmental effects, which are commonly accounted for in monitoring strategies, together with the fundamental frequency (mode 1), typically the easiest to identify, and additional frequencies exhibiting good sensitivity to damage, as quantified in Table 1. In particular, damage scenario D1 induces comparable reductions across all natural frequencies, whereas the fourth and fifth natural frequencies exhibit the largest decreases under D2. In contrast, D3 produces the most significant reductions in, in descending order, the first, fourth, and sixth natural frequencies. The selection also includes modes characterised by a high proportion of effective mass (28% and 16% in the vertical direction for modes 1 and 5, respectively, and 81% in the longitudinal direction for mode 6). Although the antisymmetric nature of mode 4 limits its effective mass, it activates a significant contribution from the lateral spans, thereby complementing the information provided by the other modes, which do not excite these portions.

Overall, this selection reflects a balance between idealised knowledge and practical constraints. While the use of artificially generated monitoring data enables a rigorous and transparent evaluation of feature sensitivity, real-world SHM applications are inherently affected by limited information, due to the restricted number and placement of sensors, as well as other factors influencing the identifiability and interpretability of structural modes. These aspects can significantly affect both the number and reliability of the features that can be monitored in practice. Accordingly, the chosen set limits the number of feature pairs to a manageable level, while remaining sufficiently comprehensive to demonstrate a methodology that is both scalable and applicable to realistic scenarios characterised by uncertainty and partial observability.

The offline training and validation stages are conducted over a two-year period. The training phase considers samples spanning one and a half years, from the beginning of 1 January 2023 to midnight of 30 June 2024, while the validation phase includes samples from 1 July 2024 to midnight on 31 December 2024. The final year of monitoring is used for testing. Self and nonself samples, in the testing phase, differ only in the introduction of damage. Thus, at each time instant, the temperature remains the same, and both the density and Young’s modulus of each model element are identical, except for the reductions associated with damage. This minimises the bias in the evaluation of the DI performance. The temperature during the training period ranges from −3 to 40 °C, while in the subsequent monitoring phase it remains within a slightly narrower interval of −2 to 39 °C. Therefore, no “out of range” issues arise due to temperature variations when MMN is adopted.

**Figure 8:**
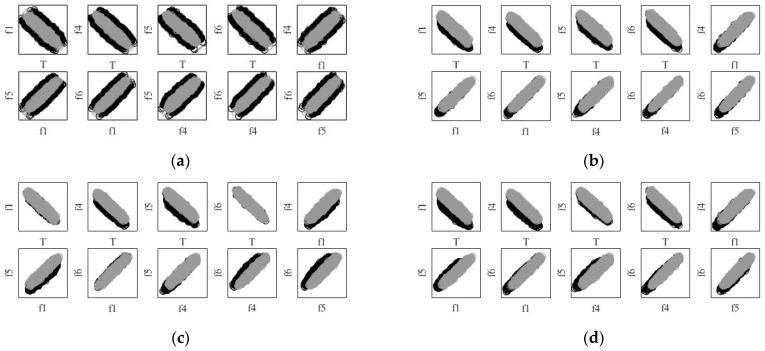
Min–max normalisation in the dimensionless unitary [0, 1]^2^ space, self samples in grey, nonself samples in black: (**a**) validation dataset; (**b**) testing D1 scenario; (**c**) testing D2 scenario; (**d**) testing D3 scenario.

**Figure 9:**
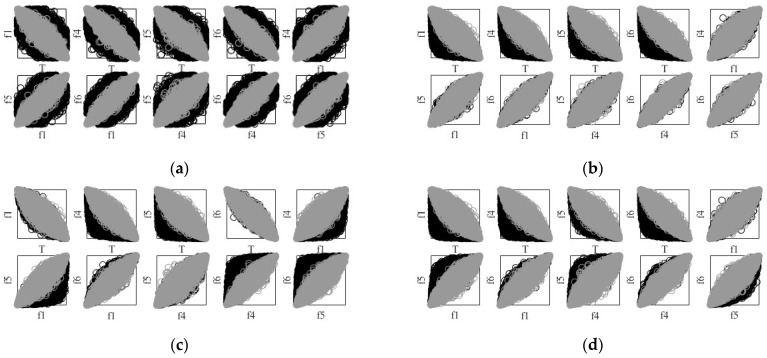
Soft max scaling in the dimensionless unitary [0, 1]^2^ space, self samples in grey, nonself samples in black: (**a**) validation dataset; (**b**) testing D1 scenario; (**c**) testing D2 scenario; (**d**) testing D3 scenario.

**Figure 10:**
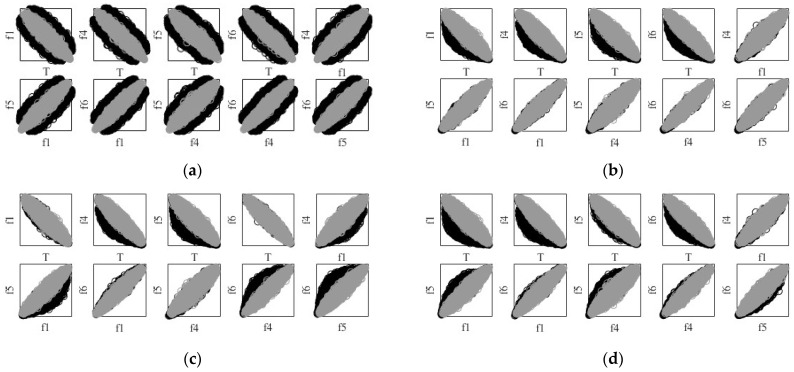
1Z-score normalisation in the dimensionless unitary [0, 1]^2^ space, self samples in grey, nonself samples in black: (**a**) validation dataset; (**b**) testing D1 scenario; (**c**) testing D2 scenario; (**d**) testing D3 scenario.

**Figure 11:**
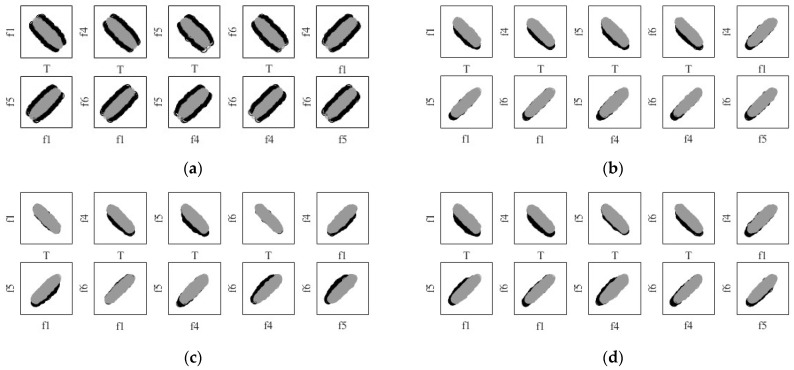
3Z-score normalisation in the dimensionless unitary [0, 1]^2^ space, self samples in grey, nonself samples in black: (**a**) validation dataset; (**b**) testing D1 scenario; (**c**) testing D2 scenario; (**d**) testing D3 scenario.

The overlap between the self and nonself samples for the validation set and the three testing sets (scenarios D1 to D3) is presented in Figure 8 for the feature spaces obtained using MMN, in Figure 9 for SMS, in Figure 10 for 1ZS and in Figure 11 for 3ZS.

**Figure 12:**
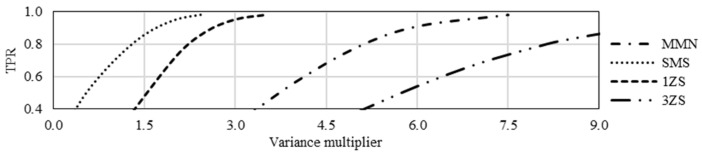
Average TPR of all classifiers based on min–max, soft max, 1Z-score and 3Z-score normalisation over the validation dataset.

These representations illustrate the effect of the strategy used to generate artificial nonself samples, as described in Section 2. Specifically, the nonself data are obtained by translating a copy of the self samples along the narrow direction of the distribution on one side, and a second copy on the opposite side. This approach effectively flanks the self samples. The magnitude of the translation is defined according to the scaling method, as this influences the variance of the feature distribution. To ensure a fair and reproducible comparison between the scaling methods considered, a quantitative optimisation criterion is introduced based on the average TPR achieved on artificial nonself validation samples across all estimated combinations. More specifically, the variance multiplier is selected such that an average TPR of 0.8 is achieved on the simulated nonself validation data, thereby ensuring adequate coverage of the nonself domain. The evolution of the TPR as a function of the variance multiplier is shown in Figure 12. The TPR threshold influences the final detection performance on the testing set. However, no reliable criterion has yet emerged to predict this influence and, consequently, optimisation of the threshold remains an important direction for future work. For MMN, SMS, 1ZS, and 3ZS, the selected translations correspond to 5.1, 1.3, 2.2, and 8.2 times the variance, respectively.

For each feature space, the validation stage aims to optimise the detector radius and the censoring distance. These are treated as dependent variables, defined respectively by the spacing between detector centres (for the radius) and by a multiplier of the detector radius (for the censoring distance). A total of 120 combinations is evaluated, comprising 20 values of the spacing (ranging from 0.005 to 0.1 in increments of 0.005) and six values of the multiplier (ranging from 1 to 2 in increments of 0.2), to identify the optimal configuration. The optimised detector radii and distance multipliers are presented in Table 2. In most cases, the validation process favours small radii and small/intermediate censoring distances. In contrast, the analysis of the best overall detector sets indicates that intermediate radii are more suitable for SMS. Large radii only yield good performance in feature spaces with low sensitivity to the specific damage scenario; however, in these cases the ROCAUC typically remains below 0.55, indicating a high rate of misclassification.

**Figure 13:**
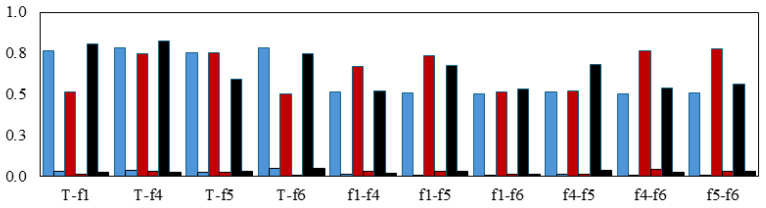
The ROCAUC, mean (longer bar) and standard deviation (shorter bar) of the optimised classifiers in different normalisation methods for the D1 (blue), D2 (red), and D3 scenarios (black).

Figure 8, Figure 9, Figure 10 and Figure 11 also allow the analysis of the sensitivity of the feature spaces to specific damage scenarios. Diffuse deterioration (D1) produces nearly uniform reductions in the natural frequencies, resulting in a translation of the features in spaces that combine pairs of natural frequencies along their principal direction. Consequently, a significant portion of nonself samples overlaps with the self region. For this scenario, feature spaces incorporating temperature present higher damage sensitivity. Under normal temperature variations, the natural frequencies decrease, so damage induces a translation of the nonself distribution along a narrower direction. Feature spaces including temperature are, on average, rather sensitive to localised damage (scenarios D2 and D3) as well. However, in these cases, the sensitivity of the two-dimensional feature spaces depends on the responsiveness of the natural frequencies. For example, D2 causes negligible variations in modes 1 and 6, while D3 has limited influence on mode 5. This damage sensitivity is confirmed irrespective of the normalisation method. The mean values across normalisation methods are shown in Figure 13, together with the standard deviation, which consistently assumes small values.

Finally, the comparison of the figures also highlights the influence of the scaling methods on the distortion of the original self and nonself feature distributions. Methods based on the mean and standard deviation introduce a rescaling of the feature space relative to its original dimensions, governed by the multiplier applied in the denominator. As this multiplier increases, from approximately 0.48 for soft max scaling to 3 for 3Z-score normalisation, the distributions tend to contract within the unit feature space. This contraction involves more the samples located closer to the mean in a region that depends on the multiplier itself, while it produces a magnification of the distance of the remaining samples. Smaller multipliers amplify deviations from the mean, thereby increasing the apparent separation between nonself samples and the self region. However, this effect is balanced by the concurrent expansion of the self region itself, particularly when it exhibits high variance.

**Figure 14:**
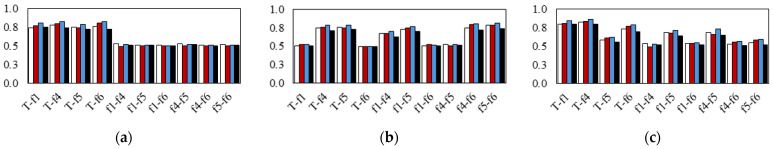
The ROCAUC optimised classifiers based on min–max (white), soft max (red), 1Z-score (blue) and 3Z-score normalisation (black): (**a**) D1 scenario; (**b**) D2 scenario; (**c**) D3 scenario.

The aforementioned distortion has a direct impact on the FPR. 3ZS consistently yields the lowest number of false positives for the optimised classifiers. MMN maintains the FPR below 1%, whereas 1ZS and SMS can reach values of up to 5% for certain feature spaces. However, this increase in FPR is balanced by a higher TPR, which is, on average, greater for optimised classifiers based on 1ZS. As a result, this approach achieves superior performance in terms of the ROCAUC. Figure 14 presents the performance of the classifiers optimised through the validation stage.

**Figure 15:**
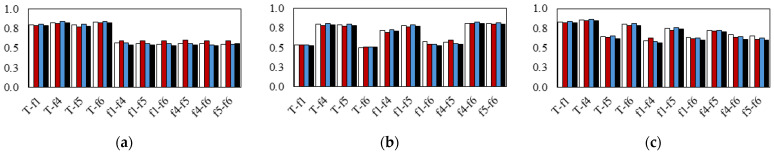
The ROCAUC best overall classifiers based on MMN (white), SMS (red), 1ZS (blue) and 3ZS (black): (**a**) D1 scenario; (**b**) D2 scenario; (**c**) D3 scenario.

Despite the enhancement of the validation dataset, to include artificial nonself samples, the optimised classifiers do not generally correspond to the best overall solution for each feature space and scaling strategy, with only a few exceptions for 1ZS normalisation. ROCAUC performance values for the best overall classifiers are presented in Figure 15. It should be noted that this comparison is complicated by the damage-specific optimality of each detector set, which results from the aforementioned peculiar sensitivity of the feature to the specific scenarios. While the optimisation based on the validation set produces a single optimal detector set for a given scaling method and feature space, the best overall solution varies depending on the actual damage scenario.

**Figure 16:**
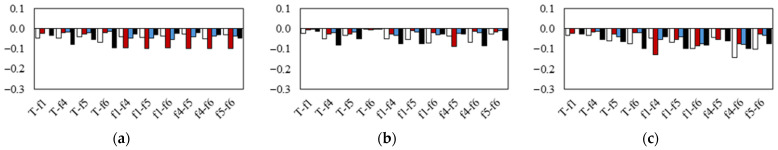
Differences in the ROCAUC between the optimised and best overall classifiers based on MMN (white), SMS (red), 1ZS (blue) and 3ZS (black): (**a**) D1 scenario; (**b**) D2 scenario; (**c**) D3 scenario.

Figure 16 illustrates the reduction in performance (in terms of the ROCAUC) of the optimised classifiers relative to the best overall classifiers for the same feature space and scaling method. On average, this performance gap is smallest for 1ZS, whereas it is larger for 3ZS in damage-sensitive feature spaces.

**Figure 17:**
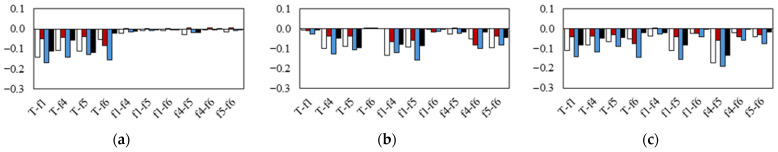
Differences in the ROCAUC between the classifiers optimised according to FPR and according to the ROCAUC in the validation stage based on MMN (white), SMS (red), 1ZS (blue) and 3ZS (black): (**a**) D1 scenario; (**b**) D2 scenario; (**c**) D3 scenario.

Nonetheless, the optimisation strategy, based on artificial nonself samples to maximise the ROCAUC, systematically yields better classifiers for damage-sensitive feature spaces than a strategy based solely on self samples and aimed at minimising the FPR. The reduction in performance during the monitoring stage when adopting classifiers optimised only for FPR is shown in Figure 17. This arises from the imbalanced optimisation criterion introduced by focusing exclusively on self samples. While this approach prevents false alarms, it produces classifiers with poorer coverage of the boundary between self and nonself regions, and therefore tends to detect damage only at a more advanced stage. Indeed, parameter settings based on minimising the FPR favour larger censoring distances, about twice the detector radius, as detectors are positioned further away from the training self samples.

**Figure 18:**
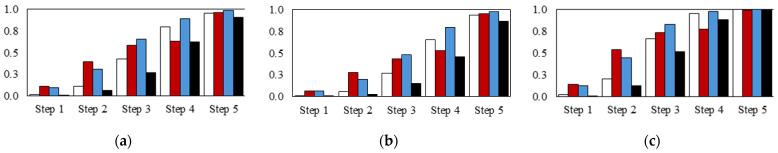

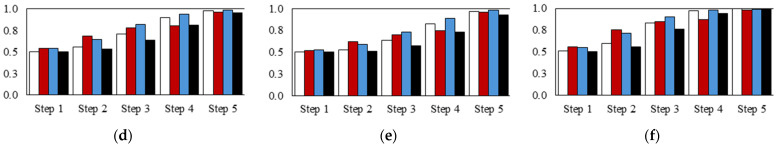
The TPR (**a**–**c**) and ROCAUC (**d**–**f**) best overall classifiers in the T-f4 feature space based on MMN (white), SMS (red), 1ZS (blue) and 3ZS (black) for each incremental step of damage: (**a**,**d**) D1 scenario; (**b**,**e**) D2 scenario; (**c**,**f**) D3 scenario.

**Figure 19:**
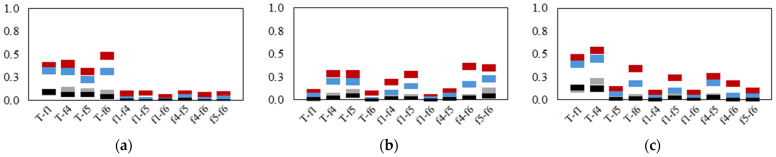
The TPR 95% confidence interval for the second step of damage best overall classifiers based on MMN (grey), SMS (red), 1ZS (blue) and 3ZS (black): (**a**) D1 scenario; (**b**) D2 scenario; (**c**) D3 scenario.

Early warning is a critical requirement for any damage detection strategy, and the choice of scaling method plays a key role in this respect. Across the three damage scenarios considered, consistent detection of positive samples begins, on average, at the third incremental step. Before this, for most optimised classifiers, the TPR is larger than the FPR, yet the percentage of positive labels is limited. Only SMS and 1ZS, likely due to the greater distortion introduced into the original distributions, yields a marked increase in the TPR as early as the second stage. Figure 18 presents both the TPR and ROCAUC for classifiers trained in the T-f4 feature space. This feature space is selected as it is sensitive to all three damage scenarios considered. However, similar trends are observed across the other feature spaces as well. The higher TPR in the second step enables identification with a sufficient level of confidence, as not only isolated and sparse samples are identified as positive, but also consecutive samples are consistently classified. Using consecutive positive classifications is, indeed, a simple and effective strategy to limit false alarms [24,45]. The reliability of the comparison of classifier responsiveness to small damage extents (second damage step, March and April 2025 data) is supported by statistical analysis. First, the McNemar test is employed to compare the classification behaviour associated with different scaling methods over undamaged and small-damage samples. Since the classifiers tend to misclassify different samples, the test confirms statistically significant differences in the detector responses among the considered scaling approaches. Furthermore, the confidence intervals for the TPR are estimated using a non-parametric bootstrap procedure. Specifically, the test set is resampled with replacement 2000 times to generate bootstrap replicas of the original dataset, and the TPR is recomputed for each sample. The empirical distribution of the bootstrap estimates is then used to derive the 95% confidence intervals by considering the α/2 and 1−α/2 quantiles, with α=0.05. No overlap is observed between the confidence intervals of the best-performing classifiers associated with the different scaling methods (Figure 19), confirming the superior performance of the classifiers normalised using SMS and 1ZS.

**Figure 20:**
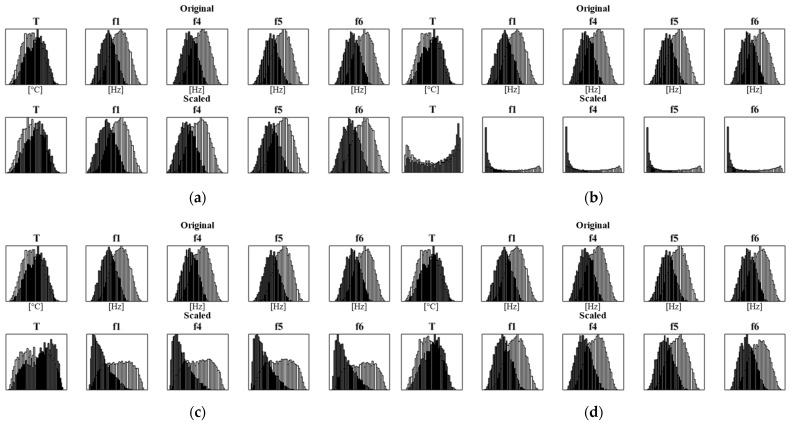
Impact of the scaling and distribution of self (grey) and nonself (black) samples in the original feature and after scaling: (**a**) MMN; (**b**) SMS; (**c**) 1ZS; (**d**) 3ZS.

In light of the above, scaling strategies that maximise the distance of samples from the region around the central tendency emerge as a promising approach for improving damage detection performance, particularly by enhancing sensitivity to small damage levels. A second advantage of such scaling methods is illustrated in Figure 20, where the distributions of self and nonself samples are compared in the original space and after scaling. As expected, MMN preserves the distribution shapes. The 3ZS approach also largely preserves the shape, as samples in the original space remain sufficiently close to the mean. In contrast, 1ZS and SMS alter the shape, effectively increasing the separation of nonself samples from the mean. For 1ZS, the self distribution remains relatively compact, which likely explains the superior overall performance of classifiers trained using this scaling method. By contrast, SMS produces a more dispersed self distribution, with several samples located in the tails. In both cases, however, the nonself distribution exhibits a higher probability of samples occurring in the tails, i.e., far from the mean of the self set.

This increased separation implies that classifiers are not only more responsive, but also less likely to encounter nonself samples close to the self region and therefore be at risk of misclassification. In this context, the TPR and, consequently, the ROCAUC derived from it, may be misleading metrics for comparing classifier performance across feature spaces, as they are computed a posteriori over the entire set of nonself samples. A conditional measure of classification performance, accounting for the probability of sample occurrence, would further favour scaling approaches that modify the original distribution shape. The development of such metrics and their implementation to enhance confidence in early warning represent a relevant direction for future work.

A final remark concerns the size of the detector set. This affects the computational cost in two respects: the storage requirements, limited to the coordinates of detector centres in the two-dimensional space, and the detection time during the testing phase, where distances between each new observation and the detectors must be computed and compared against a threshold. The results are presented in Table 3.

A larger self region in the feature space results in a reduced number of detectors required to cover the nonself space. On average, 1ZS requires the greatest number of detectors, followed by SMS. Across the 120 parameter combinations, 3ZS yields sets ranging from 83 to 38,937 detectors, the highest observed value among all scaling methods. In contrast, SMS produces, on average, the smallest detector sets, with a minimum of only 43 detectors. This trend is partially reflected in the optimised classifiers. The largest detector sets are associated with 3ZS, as expected, with an exceptionally large set for 1ZS in the *T-f*_4_ space. Conversely, the size of the optimised sets in feature spaces processed with SMS is also large, due to the predominance of very small detector radii.

## 5. Conclusions

The present work analysed the performance of NSAs for damage detection and early warning in the SHM of civil structures and infrastructure. NSAs are computationally efficient machine learning algorithms suited to one-class classification, relying solely on data representative of a reference system behaviour assumed as normal. Despite their extensive use in other fields, their application in SHM remains limited. Existing studies report promising results but give limited attention to algorithm architecture and parameter optimisation.

Building on previous works, the authors employed a Deterministically Generated NSA (DGNSA) and further investigated its capabilities by exploring the parameter space through a refined tuning procedure, and by assessing the influence of feature scaling on the definition of the feature space. An improved censoring strategy was also introduced, based on a robust criterion for detector elimination.

A controlled and replicable testbed was developed using a numerical finite element model, enabling the generation of realistic monitoring data for a reinforced concrete bridge under temperature variations across four scenarios: undamaged conditions, diffuse deterioration, and two localised damage cases.

The analyses led to the following main conclusions:

- Not all features are equally sensitive to damage. When feature pairs are used for classification, performance improves if the damage induces different variations in each feature.
- Incorporating nonself samples for parameter tuning improves coverage of the boundary between normal and anomalous behaviour. The proposed strategy for generating artificial nonself samples effectively surrounds the self region. However, it yields suboptimal classifiers compared to the best parameter combinations. This is partly because the tuning procedure produces a single classifier, whereas optimal parameter settings vary with the damage scenario.
- For the case study considered, small detector radii and small/intermediate censoring distances yield higher-performing classifiers, consistent with previous studies involving large and dense training sets. However, this finding should be generalised with caution, as it depends on the characteristics of the training data and should be validated through optimisation on a validation set as in the approach here presented.
- Feature scaling has a negligible impact on detection performance for large damage extents, where self and nonself distributions are sufficiently distinct. However, it plays a critical role in early warning for small damage extents.
- Scaling methods that amplify deviations from the central tendency of the self distribution are potentially more effective in detecting small damage, particularly when the self distribution exhibits low variance.

Due to the common nature of feature scaling and normalisation, the main results obtained regarding the influence of the scaling methods may be readily generalised to other machine learning algorithms employed for damage detection.

Coupling scaling approaches based on standardisation or reduced standard deviation multipliers λ (e.g., 1ZS or SMS) with statistical metrics that account for the probability that new samples belong to the undamaged distribution appears to be a promising strategy for reducing false alarms in the presence of small damage. In the comparison of different values of λ, a value of 0.48 demonstrated greater sensitivity to small damage extents. However, the performance assessment required an a posteriori evaluation using the testing data, which would not be feasible for the initial parameter calibration in a real-world scenario, where only the initial training data are available. Further investigation of this aspect represents a relevant direction for future work and should be extended across additional case studies to determine whether these findings can be generalised.

## Figures and Tables

**Table 1:** Maximum average frequency shift at the last damage step, considering reference conditions.

	*f*_1_	*Δf*_1_	*f*_2_	*Δf*_2_	*f*_3_	*Δf*_3_	*f*_4_	*Δf*_4_	*f*_5_	*Δf*_5_	*f*_6_	*Δf*_6_
Reference	4.05	–	10.80	–	13.74	–	15.25	–	24.29	–	31.67	–
D1	3.99	1.5%	10.64	1.5%	13.53	1.5%	15.02	1.5%	23.92	1.5%	31.19	1.5%
D2	4.04	0.4%	10.78	0.2%	13.64	0.7%	15.02	1.5%	23.86	1.8%	31.66	0.0%
D3	3.97	2.0%	10.78	0.2%	13.54	1.4%	14.96	1.9%	24.08	0.8%	31.20	1.5%

**Table 2:** Detector radius size (R) and censoring distance multiplier (DM) for the optimised detector sets.

	MMN		SMS		1ZS		3ZS	
	R	DM	R	DM	R	DM	R	DM
*T-f*_1_	0.007	1.6	0.007	1.2	0.007	1.0	0.007	1.2
*T-f*_4_	0.018	1.2	0.007	1.4	0.004	2.0	0.007	2.0
*T-f*_5_	0.007	1.2	0.007	1.4	0.007	1.2	0.007	1.4
*T-f*_6_	0.011	1.8	0.007	1.2	0.007	1.4	0.014	1.6
*f*_1_-*f*_4_	0.014	1.0	0.007	1.2	0.011	1.0	0.007	1.4
*f*_1_ *-f*_5_	0.025	1.0	0.007	1.2	0.014	1.0	0.018	1.2
*f*_1_ *-f*_6_	0.014	1.4	0.007	1.4	0.007	1.6	0.014	1.6
*f*_4_ *-f*_5_	0.011	1.0	0.007	1.2	0.007	1.0	0.011	1.0
*f*_4_ *-f*_6_	0.021	1.2	0.007	1.2	0.007	1.4	0.025	1.2
*f*_5_ *-f*_6_	0.011	1.2	0.007	1.2	0.007	1.2	0.011	1.6

**Table 3:** Detector set size and minimum and maximum values considering the parameter setting and optimised solution.

	*T-f*_1_	*T-f*_4_	*T-f*_5_	*T-f*_6_	*f*_1_-*f*_4_	*f*_1_ *-f*_5_	*f*_1_ *-f*_6_	*f*_4_ *-f*_5_	*f*_4_ *-f*_6_	*f*_5_ *-f*_6_
MMN										
Min	73	75	74	75	75	76	79	73	75	79
Max	37,813	38,086	37,825	38,094	37,931	38,138	38,220	37,900	38,264	38,122
Optimised	9037	1462	9140	3934	2291	722	2290	3988	998	4001
SMS										
Min	46	50	45	52	45	49	53	43	48	50
Max	37,660	37,558	37,619	37,525	37,260	37,086	37,062	37,247	36,920	37,240
Optimised	6893	6976	6576	7324	6821	7118	7144	6737	7416	7043
1ZS										
Min	63	65	61	67	62	66	67	59	67	65
Max	35,889	36,220	36,008	36,288	35,703	35,986	36,099	35,504	36,206	35,840
Optimised	8242	33,349	8045	8274	3449	2034	8287	8169	8324	8259
3ZS										
Min	84	84	83	85	84	84	84	84	84	84
Max	38,687	38,891	38,680	38,902	38,684	38,803	38,929	38,616	38,937	38,809
Optimised	9552	9510	9527	2377	9519	1526	2385	4147	749	4142

## Data Availability

Data will be made available on request.
